# Deep Residual Learning for Nonlinear Regression

**DOI:** 10.3390/e22020193

**Published:** 2020-02-07

**Authors:** Dongwei Chen, Fei Hu, Guokui Nian, Tiantian Yang

**Affiliations:** 1 School of Mathematical and Statistical Sciences, Clemson University, Clemson, SC 29641, USA; dongwec@clemson.edu (D.C.); tiantiy@clemson.edu (T.Y.); 2 State Key Laboratory of Atmospheric Boundary Layer Physics and Atmospheric Chemistry, Institute of Atmospheric Physics, Chinese Academy of Sciences, Beijing 100029, China; 3 College of Earth Science, University of Chinese Academy of Sciences, Beijing 100049, China; 4 State Key Laboratory of Numerical Modeling for Atmospheric Sciences and Geophysical Fluid Dynamics, Institute of Atmospheric Physics, Chinese Academy of Sciences, Beijing 100029, China; 5 Forecast Weather (Suzhou) Technology Co., Ltd., Suzhou 215000, China

**Keywords:** nonlinear regression, nonlinear approximation, deep residual learning, neural network

## Abstract

Deep learning plays a key role in the recent developments of machine learning. This paper develops a deep residual neural network (ResNet) for the regression of nonlinear functions. Convolutional layers and pooling layers are replaced by fully connected layers in the residual block. To evaluate the new regression model, we train and test neural networks with different depths and widths on simulated data, and we find the optimal parameters. We perform multiple numerical tests of the optimal regression model on multiple simulated data, and the results show that the new regression model behaves well on simulated data. Comparisons are also made between the optimal residual regression and other linear as well as nonlinear approximation techniques, such as lasso regression, decision tree, and support vector machine. The optimal residual regression model has better approximation capacity compared to the other models. Finally, the residual regression is applied into the prediction of a relative humidity series in the real world. Our study indicates that the residual regression model is stable and applicable in practice.

## 1. Introduction

Functions are common conceptions in sciences and engineering that quantify the dependence or interaction between one variable and the others. Functions are classified as linear functions and nonlinear functions from the superposition principle prospective. Linear functions are analytical, easy to analyze mathematically, and satisfy the superposition principle, while nonlinear functions are complicated and even nonanalytical. People often use linear models to approximate linear functions such as multiple linear regression [1], stepwise linear regression [2], ridge regression [3], lasso regression [4], and elastic net regression [5], which do not work for nonlinear functions. However, nonlinear functions are more common in the real world. Therefore, the approximation or regression of nonlinear functions has gained a lot of attention and is more practical and meaningful in practice [6,7,8,9,10,11,12,13,14].

One of the classical approximations is the Weierstrass approximation theorem, which shows that every continuous function defined on a close interval can be uniformly approximated as closely as desired by a polynomial function [15]. Support vector regression machine [16,17] is also a well-known nonlinear approximation technique. However, it often takes a long time if support vector regression machines are trained on large datasets, and it is difficult to choose proper kernel functions. In addition, decision tree regression [18] is widely used in practice. However, decision tree can be very non-robust, and it is NP-complete to learn an optimal decision tree where NP is the abbreviation for nondeterministic polynomial time.

Since the 1980s and 1990s, artificial neural networks have been used to approach deterministic nonlinear continuous functions based on the universal approximation theorem, which states that a neural network with a single hidden layer can approximate any continuous function with compact support to arbitrary accuracy, when the width goes to infinity [19,20,21,22]. To improve the efficiency of tuning parameters of single hidden-layer feedforward networks, [23] proposes an incremental constructive method. One may randomly choose hidden nodes and only need to adjust the output weights so that the neural network is fully automatic and works as a universal approximator. To represent multidimensional nonlinear functions by feedforward neural network, [24] investigates the training process and the network approximation properties via linear algebra, which has faster execution speeds and better generalization properties. Neural networks are also used to approximate stochastic processes. [25] reports that some classes of artificial neural networks are capable of approximating prescribed stochastic processes with arbitrary accuracy in the mean square sense, which has improved approximating capabilities of artificial neural networks. [26] considers stochastic neural networks as a generalization of usually defined neural networks and shows that stochastic neural networks are more adherent to the real stochastic world. [27] also applies stochastic neural networks to approximate nonlinear input–output random transformations.

The classic universal approximation theorem relies on “fat” neural networks with infinite widths, which means there are infinitely many neurons in the hidden layer. Nevertheless, “tall” or deep neural networks are not covered in the theorem. Recently, a popular trend in machine learning is to build deeper neural networks (the so-called deep learning), ranging from LeNet [28], AlexNet [29], VGG-Net with tens of layers [30], to GoogLeNet [31] or residual neural network (ResNet) with hundreds of layers [32]. They play a central role in the progresses of machine learning and its applications. It’s observed that deeper networks perform better and approximate nonlinear functions better. [33,34] carry out research on the approximation property of a deep neural network where rectified linear units (ReLU) are used as activation functions. Their studies show that in fully connected networks, if each hidden layer has at least + 1 neurons where is the input dimension, the universal approximation theorem holds as the depth goes to infinity. [35] also shows that a deep ResNet containing only one neuron in every hidden layer can uniformly approximate any Lebesgue integrable function in dimensions.

The traditional ResNet behaves well in images process because of local convolution kernels and deep neural networks. However, the convolution kernels have no effects on the whole sequence. Thus, it is not suitable for the regression of nonlinear functions. Based on the original structure of ResNet, we build a new neural network for nonlinear regressions. Convolutional layers and pooling layers are replaced by fully connected layers in the residual block, so that the new residual neural network is more suitable for nonlinear regressions.

This paper is organized as follows. In the methodology part, the structure of regression model and datasets are introduced. In the results part, we evaluate the approximation capacity of residual regression models by performing numerical experiments. The effects of widths and depths are taken into account. When the optimal parameters are obtained, we compare the optimal residual regression with other linear and nonlinear regression techniques. We also employ the optimal residual regression into the prediction of relative humidity series. Finally, we draw conclusions.

## 2. Methodology

### 2.1. Architecture of Deep Regression Models

**Figure 1:**
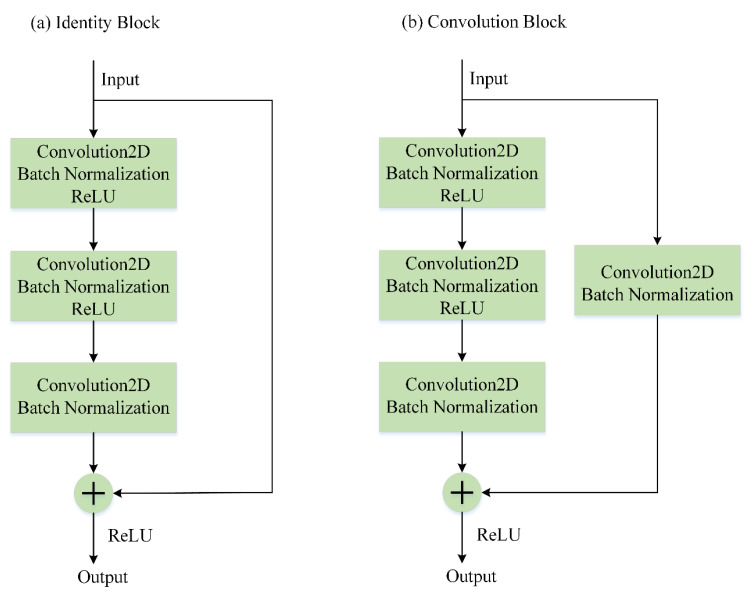
The shortcut connections of a deep residual neural network (ResNet) for the image process. (**a**) An identity block, which is employed when the input and output have the same dimensions. (**b**) A convolution block, which is used when the dimensions are different.

Residual neural network is one of the most successfully applied deep networks. [32] introduces residual shortcut connections and argues that they are indispensable for training very deep convolutional models, since the shortcuts introduce neither extra parameters nor computation complexity and increase the depth of neural network. Considering the input and output dimensions, residual shortcuts are classified as identity shortcuts and convolution shortcuts as shown in Figure 1. The identity blocks in panel (a) only require addition for tensors and can be directly used when the input and output have the same dimensions. Linear projection shortcuts are used when the dimensions are different. One linear projection shortcut consists of a 1×1 convolution unit and a Batch Normalization unit. Therefore, it is also called a convolution block as shown in panel (b). The 1×1 convolution unit can change the input dimension so that the input dimension can match with the output dimension, and then addition operations can be performed. The inputs of ResNet are often images with many channels, and it behaves well in vision imagery. However, the inputs of nonlinear functions are often one-dimensional vectors, and convolution is a not a global but rather a local operator. This means that if vectors are reshaped into matrices, the convolution kernels cannot affect the whole sequence but rather only parts of the sequence. This contradicts with the aim of nonlinear regression. Therefore, the original architecture of ResNet is not suitable for the nonlinear regression issues.

**Figure 2:**
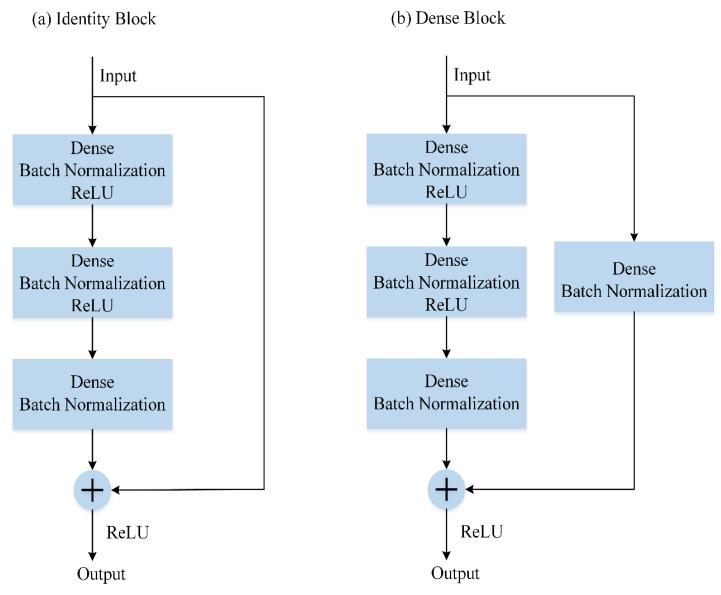
The shortcut connections of ResNet for nonlinear regression. Convolution layers are replaced by dense layers. There are three hidden dense layers in each dense block and identity block. (**a**) An identity block, which is employed when the input and output have the same dimensions. (**b**) A dense block, which is used when the dimensions are different.

Based on the structure of ResNet, we build a new neural network for nonlinear regression. Its architecture is presented in Figure 2. Convolutional layers and pooling layers are replaced by fully connected layers (or dense layers) in the residual block. Batch Normalization layers from the primary model are kept in our new model, which act as a regularizer in some cases, eliminating the need for Dropout, and allow people to use much higher learning rates and care less about initialization [36]. Panel (a) shows identity blocks that are used when the input and output are in the same dimensions. The dense blocks in panel (b) correspond to convolution blocks in Figure 1 and are used when the dimensions are different.

Usually, deep learning uses a multilayer network and employs the gradient algorithm to train models, so executing deep learning requires heavy computation, and the learning is often trapped into a saddle point or local minima [37]. To tackle this issue, people propose the rectified linear unit (ReLU) as the activation function, whose gradient can be easily computed. ReLU is shown as follows

(1) ReLU(x)=max(0,x).

In this paper, the new residual regression model employs tens or hundreds of layers. Hence, to speed up the learning convergence, ReLU is applied as activation function in hidden layers. For the output (or top) layer, linear activation function is used to meet the nonlinear regression requirement.

**Figure 3:**
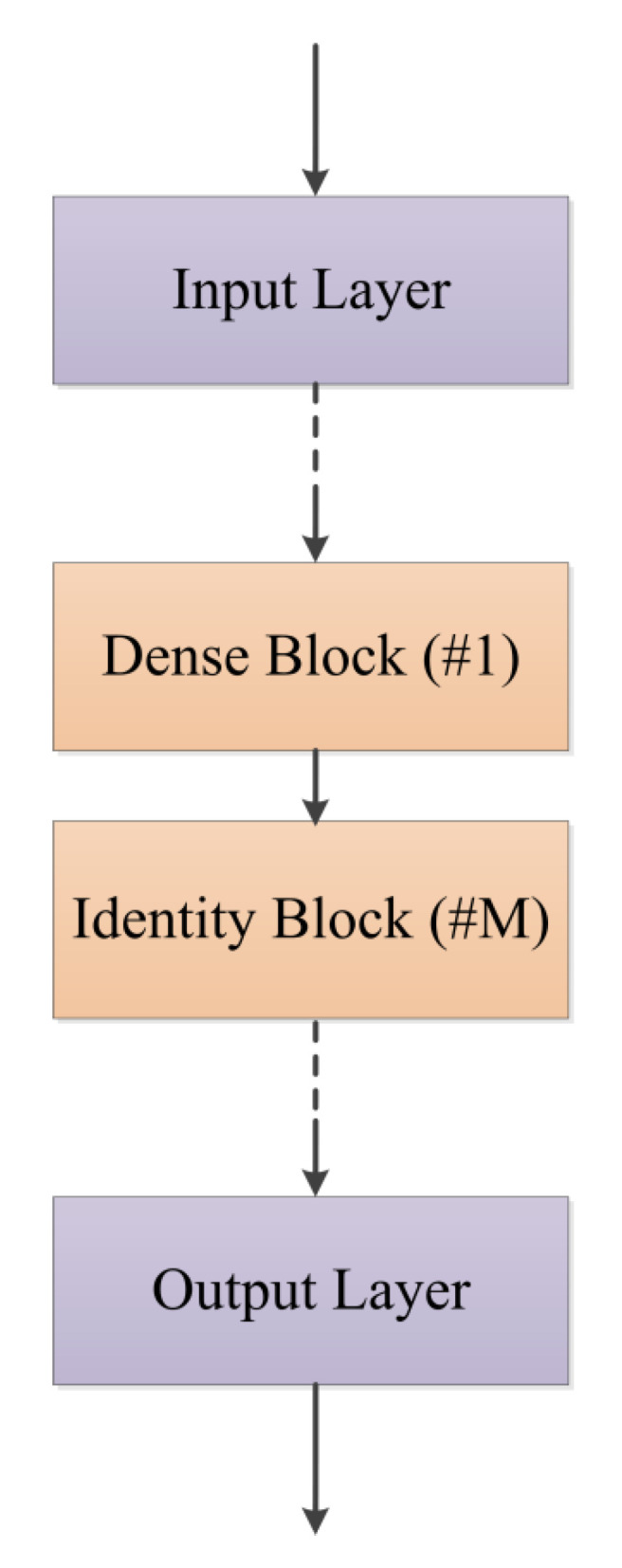
Structure of ResNet regression model. #1 means 1 dense block and #M means M identity blocks. In this paper 1 dense block and 2 identity blocks are stacked repeatedly between dashed lines.

The fundamental blocks of our model are shown in Table 1. The output dimension of the input block is equal to the input dimension of the first hidden layer. Usually, the input and output of an identity block have the same dimension (N1), but it is not true for the input dimension (N2) and output dimension (N3) of a dense block (N2≠N3). A dense block is usually inserted between two identity blocks when the output shape of one identity block is different from the input shape of the other. The structure of regression model is presented in Figure 3. The model begins with an input block and is followed by dense blocks and identity blocks. The output block is in the end. In this paper, every dense block is followed by two identity blocks, and then it is followed by one dense block, and so forth. The last two identity blocks are followed by the output layer.

### 2.2. Datasets

In this part, we introduce multiple nonlinear functions to evaluate the residual regression model. The order of nonlinearity corresponds to the number of minimum functions. Functions with the order of nonlinearity from 1 to 4 are shown as Equations (2)–(5) where $x_{i}$ is uniformly distributed in the interval [0, 4].

(2) $$y=\min\left(\overset{6}{\underset{i=0}{\sum }}{\left(x_{i}\right)}^{i},400\right),\;x_{i}\sim U\left(0,4\right),$$

(3) $$y\;y=\min\left(min\left(\overset{6}{\underset{i=0}{\sum }}{\left(x_{i}\right)}^{i},800\right),400\right),\;x_{i}\sim U\left(0,4\right),$$

(4) $$y=\min\left(min\left(\min\left(\overset{6}{\underset{i=0}{\sum }}{\left(x_{i}\right)}^{i},1200\right),800\right),400\right),x_{i}\sim U\left(0,4\right),$$

(5) $$y=\min\left(min\left(\min\left(\min\left(\overset{6}{\underset{i=0}{\sum }}{\left(x_{i}\right)}^{i},1600\right),1200\right),800\right),400\right),x_{i}\sim U\left(0,4\right).$$

**Figure 4:**
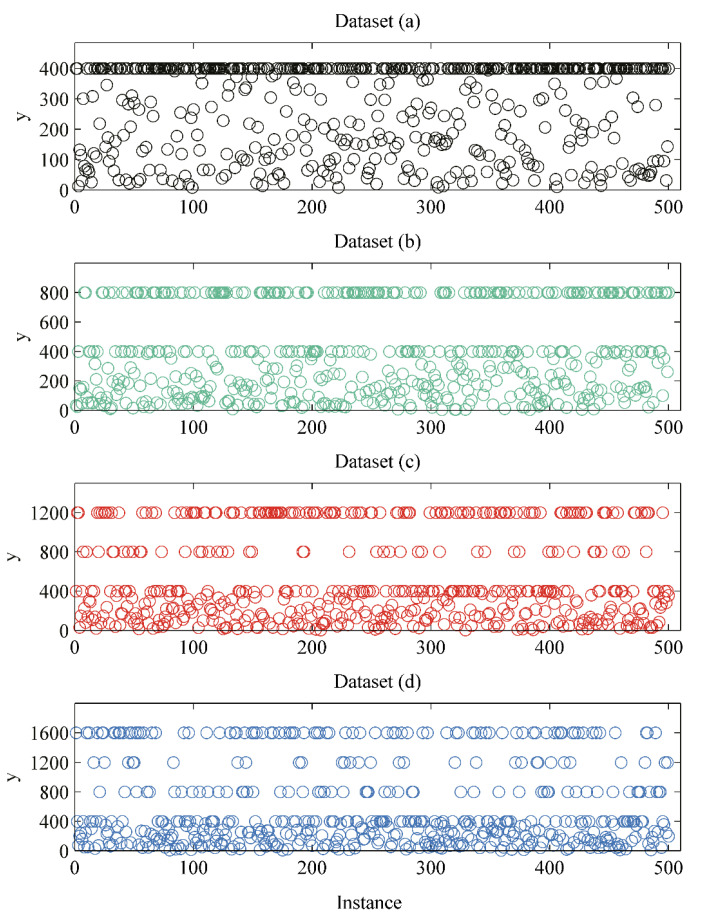
Datasets for residual regression model. (**a**), (**b**), (**c**) and (**d**) are simulated data with a nonlinearity order from 1 to 4, respectively.

In this paper, 10,000,000 samples are generated for each function. The datasets are shown in Figure 4. Panels (a), (b), (c), and (d) are samples corresponding to Equations (2)–(5) respectively. The residual regression models are trained on 6,750,000 samples and validated on 750,000 samples. The remaining 2,500,000 samples serve as testing data.

## 3. Results

### 3.1. Regression Models with Different Depths and Widths

As mentioned, the depth and width of ResNet can affect the approximation capacity. In order to evaluate the effects, we fix one factor and consider the influence of the other. Exactly speaking, when we assess the effect of depth, the width of every hidden layer is fixed, and the depth is changed. Nevertheless, when we consider the effect of width, the depth of ResNet is fixed, and the width is changed. Training data in this part are generated by Equation (4). Before training, the original data is standardized by the Min-Max scaler.

(6) $$\hat{\;w_{k}}=\frac{\underset{i}{\max}w_{i}-w_{k}}{\underset{i}{\max}w_{i}-\underset{i}{\min}w_{i}},\;$$

where $\hat{\;w_{k}}$ stands for standardized data of $w_{k}$. The residual regression models are built by Keras using TensorFlow as the backend and are trained on computer clusters with 64 CPUs and 126 GB Random Access Memory (RAM). The type of CPUs used is an Intel(R) Xeon(R) CPU E5-2683 V4 working at 2.10 GH. Every core has two threads. Training processing is also accelerated by two graphics processing units (GPUs). The type of GPUs is a GeForce GTX 1080 Ti produced by NVIDIA, and every GPU has a 10421 MB memory. We use mean squared error (MSE) as the loss function.

(7) $$loss=\frac{1}{n}\overset{n}{\underset{k=1}{\sum }}{\left(y_{k}-\hat{y_{k}}\right)}^{2}.$$

The Adam method is applied to minimize the loss function. It computes individual adaptive learning rates for different parameters [38] and combines the advantages of AdaGrad, which works well with sparse gradients [39], and RMSProp which works well in on-line and non-stationary settings [40].

An early stopping strategy is also used to avoid overfitting. When training large models, people often observe that training loss decreases over time, while validation loss begins to rise again. This means that a model with better validation loss can be obtained by returning to the time with the lowest validation loss, which is known as an early stopping strategy and is probably the most commonly used form of regularization in deep learning due to its effectiveness and simplicity [41]. The algorithm stops when no progress has been made over the best recorded validation loss for some pre-specified number (or patience) of epochs.

In this paper, the batch size for gradient descent is 5000, and the epoch number of training is 50. The patience of early stopping is 10 epochs. The train loss, validation loss, and testing loss are computed by standardized data and have the magnitude of 10^−4^. Table 2 shows the training process of regression models with different depths. Before considering the effects of depth and width, we have trained residual regression models with different widths for many times and found that testing loss is small when the width is fixed at 20. Hence by experience, the width of every hidden layer is initially fixed at 20.

From Table 2, we could see that when the depth is less than 100, testing losses range from $3.4855\times 10^{-4}$ to $5.3401\times 10^{-4}$. The testing losses are in the same magnitude and have small changes. However, when the depth is equal to or beyond 100, the testing and validation losses increase greatly. The testing losses vary from $1.8493\times 10^{-3}$ to $2.4995\times 10^{-1}$. Thus, the optimal depth of residual regression model is approximately 28, since the model has a minimum testing loss, i.e., $3.4855\times 10^{-4}$. Besides, it is not true that if the neural network is deeper, it behaves better on approximation or regression. This is because neural networks use the back propagation (BP) algorithm to minimize their loss functions, but it is difficult to optimize very deep neural networks.

Based on the optimal depth, we consider the effect of width. Table 3 shows the training information of residual regression model with different widths. The depth is fixed to the optimal depth, i.e., 28. From Table 3, we could know that the optimal width is approximately 16, since the model has a minimum testing loss. When the width changes from 8 to 700, testing losses range from $2.1360\times 10^{-4}$ to $8.1343\times 10^{-4}$, which have the same magnitude $10^{-4}$ and have small variations. Nevertheless, when the width is small (less than 4), testing and validation losses are greater than $1\times 10^{-3}$. Hence, the approximation capacities of wide neural networks are stronger than very narrow neural networks. This is because residual regression models with small widths are too simple to approximate complex nonlinear mappings. Therefore, it is not recommended to set the residual regression model with a great depth (more than 100) or a small width (less than 4).

### 3.2. Trainng the Optimal Regression Model on Nonlinear Datasets

**Figure 5:**
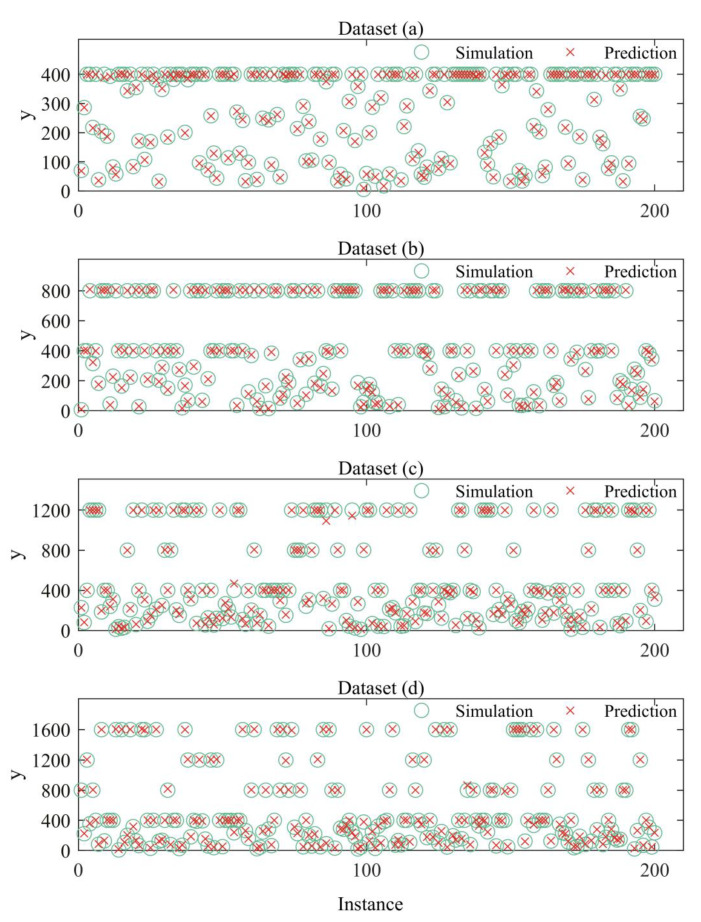
The results of optimal regression model on simulated nonlinear data. A red cross symbol (×) means predicted data and a green circle symbol (O) stands for simulated data generated by Equations (2)–(5).

Table 4 shows the training information of an optimal regression model on simulated nonlinear data. The depth is fixed at 28 and the width 16. Preprocessing of data is the same as before. From Table 4, one could see that the maxima and minima of testing losses of the optimal regression model are $4.2117\times 10^{-4}$ and $2.550\times 10^{-5}$, respectively. This indicates that the optimal model has small testing losses on these nonlinear datasets. Figure 5 shows the visualized comparisons between regression results of the optimal model and the real value of simulated datasets. For each dataset, 200 samples and the corresponding predictions are compared and plotted in Figure 5. From Table 4 and Figure 5, we could see that the optimal model behaves well in simulated nonlinear datasets.

### 3.3. Comparisons with Other Approximation Techniques

In this section, we compare the optimal residual model with other linear and nonlinear approximation techniques. Linear techniques include the linear regression, ridge regression, lasso regression, and elastic net regression, which combines ridge regression and lasso regression. Nonlinear techniques include the usual artificial neural network (ANN), decision tree, and support vector regression (SVR) machine. ANN has the same architecture (the same depth and width) as the optimal residual regression model except for the shortcut connections mentioned in Figure 2. ANN is modeled by Keras using tensorflow as a backend. Other regression models are built by the machine learning package scikit-learn in python [42]. A total of 10,000,000 samples generated by Equation (4) are used here. ANN is trained on 6,750,000 samples and validated on 750,000 samples. The remaining 2,500,000 samples are used as testing data. The epoch and patience of early warning for ANN are 50 and 10, respectively. Other models are trained on 7,500,000 samples and tested on the same 2,500,000 samples. Table 5 shows the comparison results including training time, validation loss, and testing loss. There are hyperparameters for every approximation technique excluding linear regression, which cannot be learned through training and must be set in advance. Hence, to get a good approximation, we use the grid search method to find the optimal values. Table 6 shows the information about hyperparameters, including the name, the range, and the corresponding optimal value of every hyperparameter. NA in Table 5 and Table 6 stands for Not Applicable.

From Table 5, one could observe that the testing losses of the linear models listed in the top are approximately $3.72\times 10^{-2}$, which is much greater than the testing losses of the other nonlinear approximation techniques. This indicates that these linear models are not appropriate for nonlinear approximation. It is notable that support vector regression (SVR) machines with radial basis function (RBF) kernel have the maximum training time, which is close to 44 h, and the second greatest testing loss ($1.2676\times 10^{-2}$). The testing loss of SVR is also greater than that of residual regression, ANN without shortcuts, and decision tree. These drawbacks of SVR limit its further applications in practice. It is also worthy to mention that the testing loss of decision tree regression is 4.58 times as great as that of the residual regression model. This implies that the residual regression model has better approximation capacities than decision tree. In addition, the influence of shortcut connections is investigated. ANN without shortcuts has the same depth and width as the optimal residual model, and it has the second smallest testing loss. The model is trained for nearly 27 min and early stops at 34 epochs. However, the optimal residual model is trained approximately 23 min through 50 epochs. This means that the residual regression model is more efficient and approximates nonlinear functions better.

### 3.4. Application of Residual Regression Model on Climate Data

In this paper, we employ the residual regression to approximate relative humidity. Relative humidity is defined as the ratio of the water vapor pressure to the saturated water vapor pressure at a given temperature. It is a key factor affecting cloud microphysics and dynamics, and it plays an important role in climate [43]. The formation of cloud condensation nuclei needs water vapor to be supersaturated in the air. However, currently, there is no widely accepted and reliable method to measure the supersaturated vapor pressure accurately [44], which means that the relative humidity is not accurate under supersaturation circumstance. Therefore, finding the nonlinear relationship between relative humidity and other factors is meaningful.

**Figure 6:**
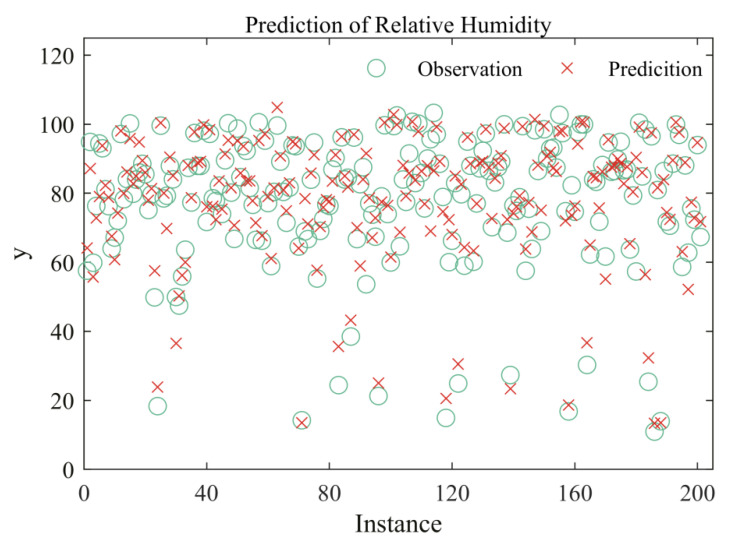
The prediction of regression models for relative humidity. The red cross symbol (×) means predicted data, and the green circle symbol (O) stands for observations of EAR5.

Our training data is from EAR5 hourly reanalysis datasets on the 1000 hPa pressure level [45]. Pressure, temperature, and specific humidity are used as input features. EAR5 is the fifth generation ECMEF (European Centre for Medium-Range Weather Forecasts) atmospheric reanalysis of the global climate, and it provides hourly outputs at a spatial resolution of 0.25°. The timestamp of the training data is from 00:00:00 to 23:00:00 on September 1, 2007. There are 24,917,709 samples in total. The optimal residual regression model is trained on 20,183,344 samples and validated on 2,242,594 samples. The remaining 2,491,771 samples are testing data. The other parameters are the same. A total of 200 observations and the corresponding predicted data are plotted in Figure 6. The relative error of testing data for relative humidity is 9%. We replace the testing dataset randomly for 10 times, and the averaged relative error is still 9%. This verifies that the residual regression model is stable and applicable in practice.

## 4. Conclusions

In this paper, we develop deep residual regression models for nonlinear regression. The traditional deep residual learning behaves well in the images process due to local convolution kernels and deep neural networks. However, the convolution kernels have no effects on the whole input sequence. Therefore, it is not suitable for the regression of nonlinear functions. We replace convolutional layers and pooling layers by fully connected layers to ensure that deep residual learning can be applied in nonlinear regression. The residual regression model is carefully and numerically evaluated on simulated nonlinear data, and the results show that the improved regression model works well. It is recommended to avoid setting the residual regression model into a great depth or a small width, since it has a great testing loss under these circumstances. In addition, we compare the residual regression model with other linear and nonlinear approximation techniques. It turns out that the optimal residual regression model has a better approximation capacity compared to others. Finally, the residual regression model is applied into the prediction of relative humidity, and we get a low relative error, which indicates that the residual regression model is stable and applicable in practice. In the future, we intend to apply the residual regression model on large eddy simulation (LES) datasets of turbulence to improve the subgrid-scale parameterizaitons of LES.

## 5. Patents

There aren’t any patents resulting from the work reported in this manuscript.

## Figures and Tables

**Table 1:** Fundamental blocks of ResNet regression.

Type of Block	Input Shape	Output Shape	Activation Function	Input and Output Are of Same Size
Input	(1, 6)	(1, None *)	ReLU	False
Dense	(1, *N*2)	(1, *N*3)	ReLU	False
Identity	(1, *N*1)	(1, *N*1)	ReLU	True
Output	(1, None)	(1,1)	Linear	False

* None means the size of input in this dimension is uncertain.

**Table 2:** Residual regression model with different depths.

Depth of ResNet	Number of Parameters	Stopping Epoch	Training Time	Training Loss (10^−4^)	Validation Loss (10^−4^)	Testing Loss (10^−4^)
10	4581	50	00:30:04	6.6637	3.6566	3.6687
19	9581	37	00:23:42	6.7196	4.4022	4.3824
28 *	**14,581**	**44**	**00:55:34**	**5.7221**	**3.5205**	**3.4855**
37	19,581	50	00:59:48	5.0877	5.1125	5.2183
46	24,581	34	01:16:57	6.3228	5.3790	5.3401
55	29,581	41	02:14:36	5.4676	4.3013	4.1437
82	44,581	44	02:27:26	6.6924	3.8426	3.8526
100	54,581	50	02:25:18	8.2667	19.000	18.943
145	79,581	39	03:46:58	15.000	2497.0	2499.5
244	134,581	50	09:09:05	1273.0	1277.0	1279.0

* The bold implies that the depth of ResNet is optimal and has a minimum testing loss.

**Table 3:** Residual regression model with different widths.

Width of ResNet	Number of Parameters	Stopping Epoch	Training Time	Training Loss (10^−4^)	Validation Loss (10^−4^)	Testing Loss (10^−4^)
1	198	50	00:41:38	48.000	547.00	548.44
4	1125	50	00:44:36	15.000	13.000	13.255
8	3145	50	00:44:13	5.6651	3.0078	3.0666
12	6061	50	00:20:54	5.2821	6.7455	6.6151
16 *	**9873**	**50**	**00:22:22**	**4.7534**	**2.1222**	**2.1360**
20	14,581	44	00:55:34	5.7221	3.5205	3.4855
30	30,271	50	00:54:05	4.8670	3.5846	3.5973
50	78,451	42	00:18:28	5.5210	3.9034	3.9000
70	149,031	44	00:21:20	5.1793	4.2886	4.2837
90	242,011	50	00:22:11	4.5157	2.8237	2.8627
150	655,351	50	01:02:57	5.4313	3.4565	3.4189
300	2,570,701	47	00:45:00	5.6756	8.2258	8.1343
500	7,084,501	50	01:16:35	6.2678	4.0093	3.9767
700	13,838,301	50	01:56:21	6.8682	3.3580	3.4051

* The bold implies that the width of ResNet is optimal and has a minimum testing loss.

**Table 4:** Optimal regression model on nonlinear datasets.

Nonlinearity of Dataset	Number of Parameters	Stopping Epoch	Training Time	Training Loss (10^−4^)	Validation Loss (10^−4^)	Testing Loss (10^−4^)
1	9873	45	00:46:15	1.0619	0.2552	0.2550
2	9873	35	00:36:09	8.2566	4.2014	4.2117
3	9873	50	00:22:22	4.7534	2.1222	2.1360
4	9873	50	00:57:26	3.6542	2.0439	2.0481

**Table 5:** Comparisons of regression techniques.

Regression Techniques Used	Stopping Epoch	Training Time	Training Loss (10^−4^)	Validation Loss (10^−4^)	Testing Loss (10^−4^)
Linear regression	NA	00:00:20	371.61	NA	371.68
Ridge regression	NA	00:00:33	371.64	NA	371.59
Lasso regression	NA	00:00:39	371.51	NA	371.97
Elastic regression	NA	00:34:48	371.51	NA	372.00
Residual regression *	**50**	**00:22:22**	**4.7534**	**2.1222**	**2.1360**
ANN without shortcuts	34	00:27:07	7.2640	6.6379	6.6537
Decision tree regression	NA	00:17:06	9.4083	NA	9.7777
Support vector regression	NA	43:51:36	126.75	NA	126.76

* The bold implies that the residual regression model has a minimum testing loss.

**Table 6:** Optimal hyperparameters of regression techniques.

Regression Techniques Used	Name of Hyperparameters	Range of Hyperparameters	Optimal Hyperparameters
Linear regression	NA	NA	NA
Ridge regression	Penalty parameter of $L^{2}$ norm α;	10^−10^, 10^−^^9^, 10^−^^8^ …, 10^9^, 10^10^;	10^2^
Lasso regression	Penalty parameter of $L^{1}$ norm α;	10^−^^10^, 10^−^^9^, 10^−^^8^ …, 10^9^, 10^10^;	10^−^^5^
Elastic regression	Penalty parameter α; Ratio of $L^{1}$ norm ρ;	10^−^^10^, 10^−^^9^,…, 10^10^; 0.0, 0.1,…,0.9, 1.0;	10^−^^4^; 1.0;
Residual regression	Width; Depth;	NA	16; 28;
ANN without shortcuts	Width; Depth;	NA	16; 28;
Decision tree regression	Maximum depth;	1, 2,…,9, 10;	10
Support vector regression	Penalty parameter α; RBF kernel parameter γ; Epsilon-tube parameter ε; Maximum iteration N;	10^2^, 10^3^,…, 10^7^; 10^−4^, 10^−3^,…, 10^1^; 0.1; 5000;	10^2^; 10; 0.1; 5000;
